# Reverse Transcription-Loop-Mediated Isothermal Amplification-CRISPR-Cas13a Technology as a Promising Diagnostic Tool for SARS-CoV-2

**DOI:** 10.1128/spectrum.02398-22

**Published:** 2022-09-28

**Authors:** Concha Ortiz-Cartagena, Laura Fernández-García, Lucia Blasco, Olga Pacios, Inés Bleriot, María López, Rafael Cantón, María Tomás

**Affiliations:** a Translational and Multidisciplinary Microbiology (MicroTM), Biomedical Research Institute A Coruña (INIBIC), Microbiology Department, Hospital A Coruña (CHUAC), University of A Coruña (UDC), A Coruña, Spain; b Spanish Network for Research in Infectious Diseases (REIPI) and CIBER de Enfermedades Infecciosas (CIBERINFEC), Instituto de Salud Carlos III, Madrid, Spain; c Servicio de Microbiología, Hospital Universitario Ramón y Cajal and Instituto Ramón y Cajal de Investigación Sanitaria (IRYCIS), Madrid, Spain; University of Georgia

**Keywords:** COVID-19, SARS-CoV-2, RT-LAMP, CRISPR-Cas13, CRISPR-Cas, diagnosis

## Abstract

At the end of 2019, a new coronavirus, severe acute respiratory syndrome coronavirus 2 (SARS-CoV-2), caused a pandemic that persists to date and has resulted in more than 6.2 million deaths. In the last couple of years, researchers have made great efforts to develop a diagnostic technique that maintains high levels of sensitivity and specificity, since an accurate and early diagnosis is required to minimize the prevalence of SARS-CoV-2 infection. In this context, CRISPR-Cas systems are proposed as promising tools for development as diagnostic techniques due to their high specificity, highlighting that Cas13 endonuclease discriminates single nucleotide changes and displays collateral activity against single-stranded RNA molecules. With the aim of improving the sensitivity of diagnosis, this technology is usually combined with isothermal preamplification reactions (SHERLOCK, DETECTR). Based on this, we developed a reverse transcription-loop-mediated isothermal amplification (RT-LAMP)-CRISPR-Cas13a method for SARS-CoV-2 virus detection in nasopharyngeal samples without using RNA extraction that exhibits 100% specificity and 83% sensitivity, as well as a positive predictive value (PPV) of 100% and negative predictive values (NPVs) of 100%, 81%, 79.1%, and 66.7% for cycle threshold (*C_T_*) values of <20, 20 to 30, >30 and overall, respectively.

**IMPORTANCE** The coronavirus disease 2019 (COVID-19) crisis has driven the development of innovative molecular diagnosis methods, including CRISPR-Cas technology. In this work, we performed a protocol, working with RNA extraction kit-free samples and using RT-LAMP-CRISPR-Cas13a technology; our results place this method at the forefront of rapid and specific diagnostic methods for COVID-19 due to the high specificity (100%), sensitivity (83%), PPVs (100%), and NPVs (81% for high viral loads) obtained with clinical samples.

## INTRODUCTION

Since their emergence at the beginning of the 21st century, coronaviruses have been recognized as a health concern because of their ability to cause severe respiratory infections in humans. At the end of 2019, a new coronavirus appeared, severe acute respiratory syndrome coronavirus 2 (SARS-CoV-2), producing a novel illness, coronavirus disease 2019 (COVID-19), and showing two remarkable characteristics: the virus causes the development of an unusual viral pneumonia, and it is highly transmissible and thus spreads rapidly (1–3). This led to the SARS-CoV-2 pandemic, which persists to date and has caused more than 6.2 million deaths (WHO COVID-19 Dashboard [https://covid19.who.int/]).

Fortunately, vaccination campaigns have decreased the incidence of COVID-19 (4). However, specialists claim that this virus is likely to coexist with us for a long time, as the price of vaccines and the necessary cold-chain stability make it difficult for the vaccine to reach the most remote places in the world, as SARS-CoV-2 does. Together with the fact that no efficient therapy has been developed for COVID-19, this indicates that accurate and early diagnosis in point-of-care (POC) testing is required to minimize the prevalence of SARS-CoV-2 infection (1–3).

In the last couple of years, researchers have made great efforts to develop a diagnostic technique that maintains high levels of sensitivity and specificity, without the need for expensive equipment or highly trained personnel for its implementation. Such a diagnostic technique would allow the detection of SARS-CoV-2 infection in health centers, as well as at home or in the field, which would accelerate the identification of infected patients, enabling prompt treatment and halting the spread of SARS-CoV-2 worldwide (5).

The use of nucleic acids as biomarkers has become the diagnostic gold standard, because of the species specificity of the technique and because DNA and RNA can be amplified (6).

Although the reverse transcription-PCR (RT-PCR) assay is routinely used as the gold standard diagnostic test for COVID-19 (5, 7–10), throughout the pandemic period, it has shown sensitivity levels of 45% to 60% (10) and even lower than 40%, according to some authors (7), and worrying false-negative rates of 2% to 29% (10, 11). Additional downsides of this amplification method are the elevated costs (expensive equipment for implementation and readout of results), the need for specialized personnel in laboratories and the time required (4 to 6 h) (5, 8, 10). Consequently, isothermal amplification reactions are becoming especially important in the diagnosis of COVID-19 (5). Although different methods of isothermal amplification are available, recombinase polymerase amplification (RPA) and loop-mediated isothermal amplification (LAMP) reactions are the methods most commonly used in research. The LAMP-based technique has displayed greater specificity than RPA (5, 12). LAMP has previously been used to detect several microorganisms, and the aforementioned advantages led to its optimization for COVID-19 diagnosis, and it has been applied in association with other techniques which increase diagnostic specificity, such as clustered regularly interspaced short palindromic repeat (CRISPR)-associated protein (CRISPR-Cas) systems (5, 13–15).

Naturally, CRIPSR-Cas systems provide adaptive immunity for bacteria and archaea, as they collect genomic fragments (spacers) from foreign elements (bacteriophages, plasmids, and other mobile genetic elements) that are expressed in an RNA molecule form (crRNA) that guides an endonuclease protein (Cas) to the pathogen for the final degradation of its nucleic acid material (16, 17).

Since their discovery, CRISPR-Cas systems have revolutionized the field of molecular biology. Initially, they were presented as highly specific tools for genome editing. However, they are also applicable for the diagnosis and treatment of infectious diseases and are now considered key for development in these areas (16, 17).

Class 2 CRISPR-Cas systems have a simpler effector structure, which makes them more attractive for use in genome editing, diagnosis, and treatment. In this class, Cas12 and Cas13 proteins display nonspecific endonuclease activity when activated (collateral activity) against single-stranded DNA (ssDNA) and RNA (ssRNA), respectively. This feature could be applied in clinical diagnosis, taking advantage of the reporter molecule target of this activity (collateral-based detection), which acts by amplifying the detection signal. Therefore, Cas12 and Cas13 are proposed as the most promising tools for use in diagnostic techniques, with the latter being particularly important in terms of specificity, as it has the ability to discriminate single nucleotide changes (16).

**FIG 1:**
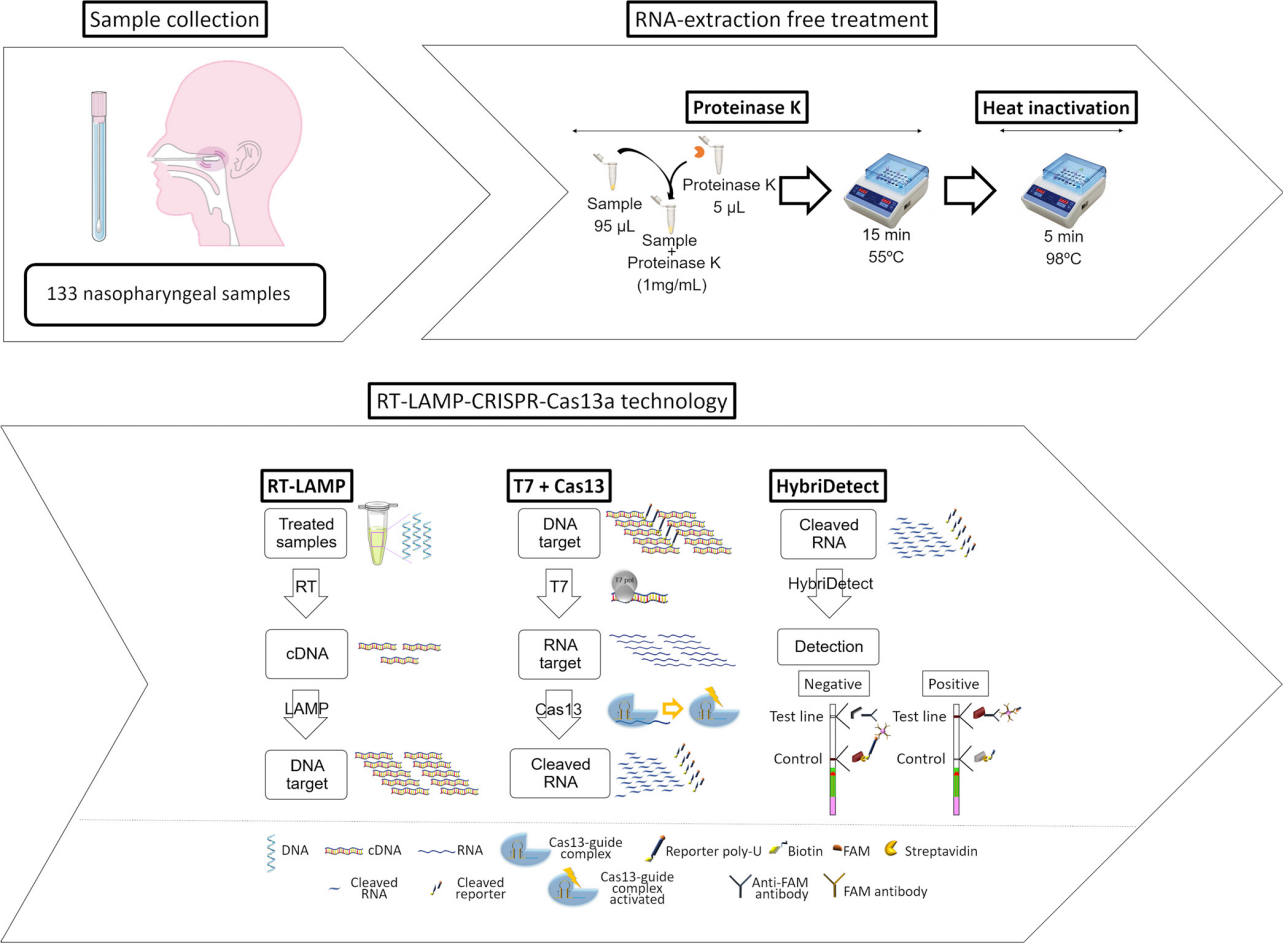
Workflow of the novel developed and optimized protocol for infectious disease diagnosis based on CRISPR-Cas13a technology.

Researchers recently developed two novel assays for detecting SARS-CoV-2 based on CRISPR-Cas technology: DETECTR and SHERLOCK. The DETECTR technique uses reverse transcription-LAMP (RT-LAMP) for amplification and Cas12 as an endonuclease, while SHERLOCK uses RT-RPA for amplification and Cas13 (18, 19). On the basis of these works, in this study, we describe the development and optimization of a LAMP-CRISPR-Cas13a technique for the diagnosis of SARS-CoV-2 infection in clinical samples in a process that does not require RNA extraction or purification (Fig. 1). With this technique, high levels of sensitivity and specificity, comparable to those associated with RT-PCR, were obtained.

## RESULTS

### Analysis of the state of the art.

We obtained an output of more than 7,000 articles as a result of a search using the keywords “RT-PCR diagnosis COVID-19”, of which almost 4,000 were published in 2021 alone. This result was compared with the findings of Bhatt et al. (20) concerning papers related to RT-LAMP and CRISPR for SARS-CoV-2 diagnosis. Of these, we analyzed 10 articles on the RT-LAMP technique and 10 articles related to RT-LAMP-CRISPR-Cas technology, finding that only 1 applied the endonuclease Cas13 for SARS-CoV-2 diagnosis, but always on samples treated with an RNA extraction kit (21) (Table 1).

**TABLE 1:** Data and parameters collected from 10 articles applying different methods to detect SARS-CoV-2 infection^*a*^

Method	Sample	RNA extraction kit	Cas	Sensitivity (%)	Specificity (%)	PPV (%)	NPV (%)	Ref.
RT-LAMP	Saliva	No		94.3	100	100	92.6	22
	NPS/OPS	No		95.2	100	100	92.6	23
	NPS	Yes		81.8	100	100	95.2	32
	NPS	Yes		94.5	99	98.8	95.2	25
	NPS/Saliva	Yes		85.9	99.5	96.8	97.4	26
	NPS	Yes		98.1	36.4	93.7	66.7	27
	NPS	Yes		98	90.9	87.5	98.6	28
	NPS/OPS/Saliva	Yes		97.8	99.9	99.8	99.9	29
	NPS	Yes		94.1	60.5	86.7	78.8	30
	NPS/Saliva	Yes		87	98.5	97.9	90.2	31
RT-LAMP-CRISPR	NPS/OPS	Yes	Cas13	97.4	100	100	66.7	34
	NPS/OPS	No	Cas12	89.7	100	100	78.6	24
	Respiratory	Yes	Cas12	94	100	100	94.3	35
	Saliva	Yes	Cas12	87.7	100	100	73.6	36
	NPS/OPS	Yes	Cas12	85.7	100	100	50	37
	NPS	Yes	Cas12	93.1	98.5	98.4	93.4	38
	Respiratory	Yes	Cas12	92.6	100	100	93.1	39
	NPS	Yes	Csm complex	73.9	100	100	45.5	21
	NPS	Yes	Cmr complex	77.5	100	100	52.6	33
	NPS	Yes	Cas3	90	95.2	90	95.2	40

a NPS, nasopharyngeal swab; OPS, oropharyngeal swab.

Data collected from the RT-LAMP articles were used to determine the range of values of the parameters considered: sensitivity, 81% to 98%; specificity, 36% to 100%; positive predictive value (PPV), 86% to 100%; and negative predictive value (NPV), 78% to 99% (Table 1). The results showed that major efforts have been made to detect SARS-CoV-2 in RNA-purified samples (8/10), although RNA extraction-free research has also yielded potentially useful results (sensitivity, >94%; specificity and PPV, 100%; NPV, >92%). However, the highest levels of sensitivity and specificity were obtained in projects involving extracted viral RNA (Table 1).

Most of the reviewed papers (8/10) related to RT-LAMP-CRISPR-Cas technology used samples treated with extraction kits. Moreover, only 1 study applied the Cas13 enzyme as an effector protein and used RNA extracted using a kit. In this case, the values for the calculated data were 73% to 97% for sensitivity, 95% to 100% for specificity, 90% to 100% for PPV, and 50% to 95% for NPV (Table 1).

### SARS-CoV-2 detection.

The best results for collateral-based detection reaction were achieved with 50 nM Cas13a enzyme and a Cas13a/crRNA molar ratio of 2:1. On the other hand, the HybriDetect lateral flow kit showed higher sensitivity when reporter 2 was used at a final concentration of 1,000 nM and the assay buffer was supplemented with 5% polyethylene glycol (PEG).

**FIG 2:**
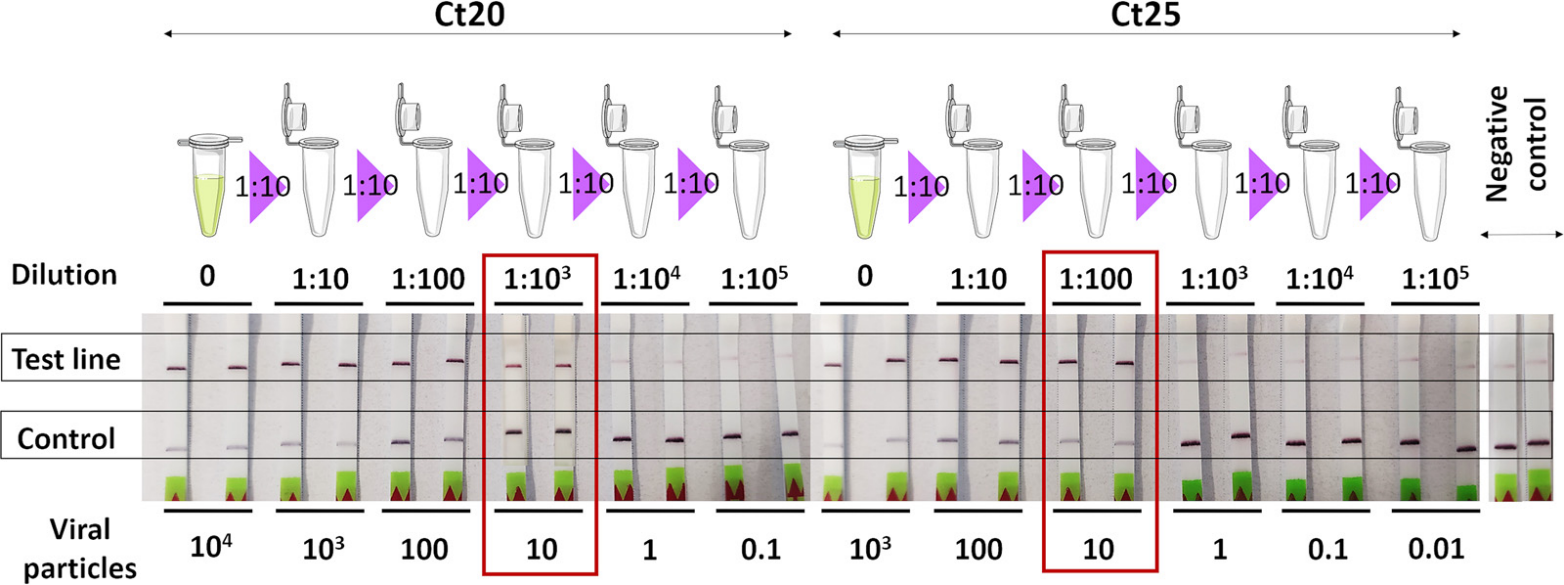
LOD assay for SARS-CoV-2 detection with the N2 gene as the target using serial dilutions (1:10) from two samples with different *C_T_* values.

**FIG 3:**
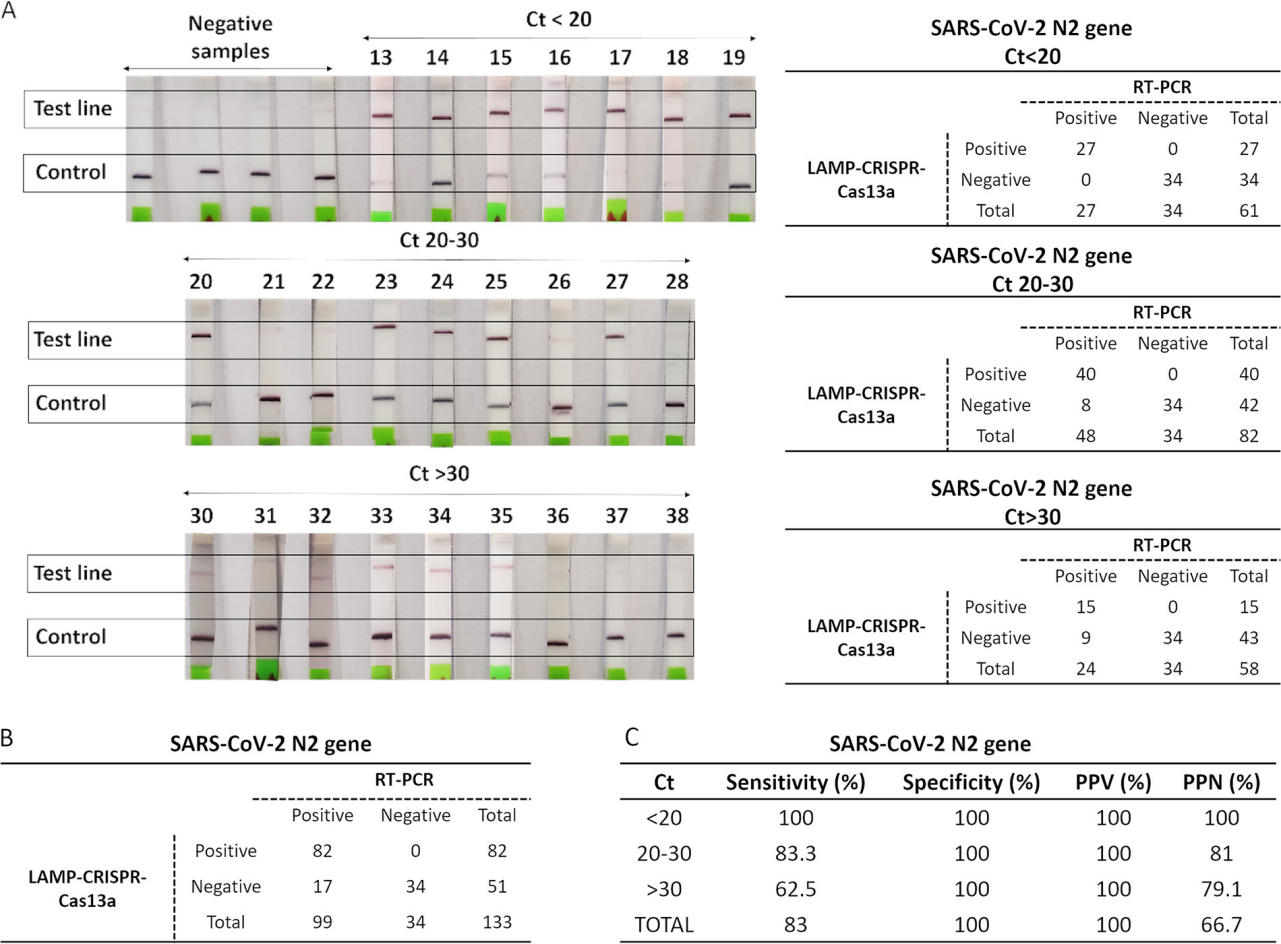
(A) Test strips (left) for SARS-CoV-2 detection using samples with *C_T_* values ranging from 13 to 38 and negative samples as negative controls, with numerical results (right) for each interval of *C_T_* values (<20, 20 to 30, and >30). (B) Results obtained using the N2 gene for SARS-CoV-2 detection. (C) Table containing the specificity, sensitivity, PPV, and NPV RT-LAMP-CRISPR-Cas13a technique values obtained by processing the data in Fig. 4B.

**FIG 4:**
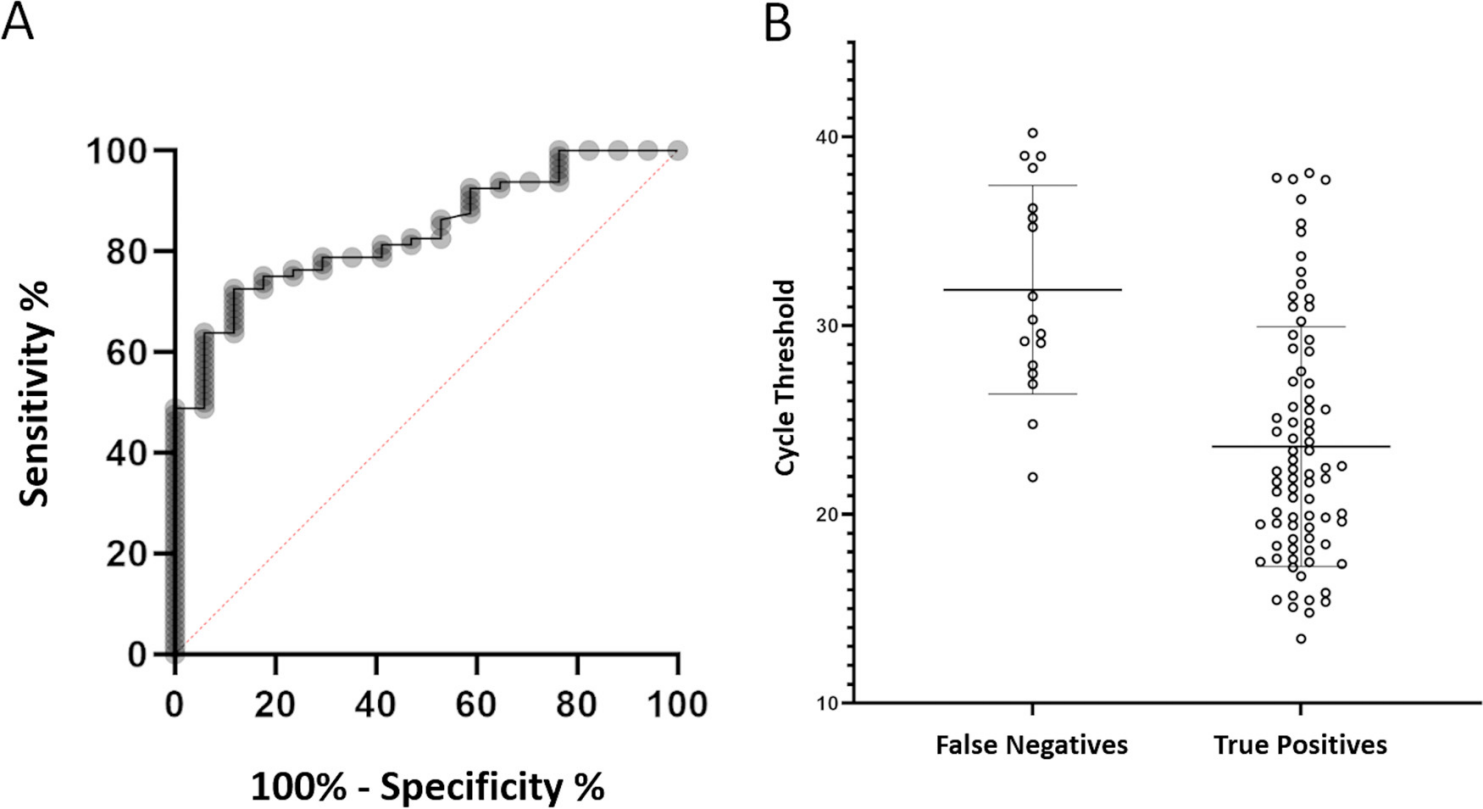
(A) ROC curve for RT-LAMP-CRISPR-Cas13a technology. (B) Scatterplot of two groups, false-negative and true-positive detections with RT-LAMP-CRISPR-Cas13a, versus the *C_T_* values of the respective samples.

Determination of the limit of detection (LOD) of the CRISPR-Cas13a-based technology revealed that this technique detects as few as 1 to 10 SARS-CoV-2 particles (Fig. 2). After proteinase K-heat inactivation (PK-HID) treatment, the LAMP-CRISPR-Cas13a technique correctly detected samples with a cycle threshold (*C_T_*) value of <20 as positive. From samples with *C_T_* values of 20 to 30 and >30, the technique identified coronavirus as present in 83.3% and 62.5% of the samples, respectively (Fig. 3C). Finally, the CRISPR-Cas13a technology did not detect SARS-CoV-2 infection in negative samples (Fig. 3A). Based on the results obtained (Fig. 3B), we estimated that the RT-LAMP-CRISPR-Cas13a method for COVID-19 detection exhibits 100% specificity and 83% sensitivity, as well as a PPV of 100% and NPVs of 100%, 81%, 79.1%, and 66.7% for *C_T_* values of <20, 20 to 30, >30 and overall, respectively (Fig. 3C). The statistical analysis yielded a receiver operating characteristic (ROC) curve with an area under the curve (AUC) of 0.84 (95% confidence interval [CI], 0.73 to 0.93) (Fig. 4A); in addition, examination of the scatterplot revealed that diagnostic results could be confused in nasopharyngeal samples with a *C_T_* value of >30 (Fig. 4B).

## DISCUSSION

Study of the state of the art revealed that greater efforts must be made to innovate in diagnostic methods; Bhatt et al. (20) found 1,286 papers related to RT-LAMP and CRISPR for SARS-CoV-2 diagnosis (surprisingly, only 98 of these applied RT-LAMP integrated with CRISPR-Cas technology), in contrast with the 7,000 studies involving RT-PCR. This indicates that efforts should also be focused on developing more efficient RT-LAMP-CRISPR-Cas protocols without RNA purification, which would reduce the cost of the testing and also produce results faster. Only 3 of the 20 papers reviewed did not use an RNA extraction kit (22–24). In addition, there are several advantages to the application of Cas13 endonuclease, as it has been reported to be more specific than other effector proteins (16).

In this work, our research group developed an RT-LAMP-CRISPR-Cas13a protocol for diagnosing SARS-CoV-2 infection with an LOD of 10 viral copies, which is similar to the LOD of the RT-PCR method, considered the gold standard for diagnosis of COVID-19 (5, 7–10, 41). However, it has been reported that the RT-PCR for SARS-CoV-2 detection has a limited sensitivity of 45% to 60% (10), while the RT-LAMP-CRISPR-Cas13a technology increases this value significantly, up to 83%. As previously mentioned, the gold standard shows downsides in terms of costs, implementation, and time consumption (5, 8, 10) that are surpassed by the RT-LAMP-CRISPR-Cas13a technique. (i) RT-PCR requires a high-quality RNA extraction method, while our technology is applied on samples processed using the simple PK-HID protocol. (ii) The gold standard depends on expensive equipment and specialized personnel, which raise the price per reaction and difficulty of use outside of laboratories; by contrast, the RT-LAMP-CRISPR-Cas13a protocol eliminates the need for a thermocycler and sophisticated readout equipment, allowing easier implementation. (iii) The RT-PCR protocol takes at least 4 to 6 h, in contrast with the RT-LAMP-CRISPR-Cas13a method, which takes less than 2 h. For all these reasons, this RT-LAMP-CRISPR-Cas13a-based assay is proposed as a strong option to replace the current molecular gold standard diagnostic test.

Furthermore, considering the criteria recommended by the WHO (42), this novel technique fulfills the three key features of accuracy, accessibility, and affordability. This is because on the one hand, it showed an accuracy [(true positive {TP} + true negative {TN})/total] of 87.2%, and on the other hand, it is both accessible and affordable.

Comparing our results on sensitivity, specificity, PPV, and NPV with those obtained in previous studies, we found that the specificity and PPV values of the RT-LAMP-CRISPR-Cas13a technology were higher than those in 7 of the 10 RT-LAMP papers reviewed (25–31), and in one case, the sensitivity of this novel technique was even higher (32). Moreover, this technique showed higher sensitivity and NPV values than those in 2 of the 10 RT-LAMP-CRISPR papers reviewed which applied an RNA extraction kit to the clinical samples (21, 33). Among the others, 7 of 8 studies used DNA target-endonuclease effectors, and thus, a higher sensitivity could be obtained due to the intrinsic stability of DNA in contrast to that of RNA molecules. The lower sensitivity of the RT-LAMP-CRISPR-Cas13a protocol (83%) than that described in a previous study (97.4%) could be explained by the fact that the researchers used an RNA extraction kit (Direct-zol), so that the RNA was purified and concentrated, and also that the results were revealed by fluorescence (34).

ROC analysis has become a popular method for evaluating the accuracy of medical diagnostic systems, as it provides accurate indices for the techniques tested that are not distorted by fluctuations caused by the use of arbitrarily chosen decision criteria or cutoff points (43). The AUC determines the inherent ability of the test to correctly identify a person as infected or not, where an AUC value of 0.5 indicates an absence of capacity for discrimination between infected and healthy populations, a value of 0.5 to 0.7 is related to unsatisfactory discrimination, and the discrimination power is acceptable when the AUC value is between 0.7 and 0.8, excellent for values contained in the range 0.8 to 0.9, and perfect when the AUC is close to 1 (44). The value of the area under the ROC curve, calculated by statistical analysis, validated our RT-LAMP-CRISPR-Cas13a technique as a reliable diagnostic method. Furthermore, the results shown in Fig. 4B indicate that this protocol provides less accurate diagnostics when viral loads are low. However, we should bear in mind that at this stage of infection, individuals present almost no risk of being contagious (45, 46).

In summary, the high levels of specificity, sensitivity, PPV, and NPV obtained using this promising protocol working with RNA extraction kit-free samples place the LAMP-CRISPR-Cas13a technology at the forefront of rapid and specific diagnostic methods for infectious diseases. Thus, this technique could be established as a diagnostic tool for detecting other viral (papillomavirus [47, 48], Zika virus [49, 50], dengue virus [50], African swine fever virus [51], Ebola virus [52]) and bacterial (53, 54) (tuberculosis [55]) diseases, as previously done by other authors for infections such as those caused by multiresistant pathogens (56, 57). However, Cas13 detection methods should be optimized to enable direct diagnosis without prior amplification of nucleic acids.

## MATERIALS AND METHODS

### Study of the state of the art.

A study of the state of the art was conducted with the aim of comparing the use of different novel diagnostic techniques. First, we conducted a search in PubMed with the keywords “RT-PCR diagnosis COVID-19” and compared the output with the number of publications on RT-LAMP and RT-LAMP-CRISPR strategies for COVID-19 diagnosis (20). Then, we collected data on the different sensitivity, specificity, positive predictive value (PPV), and negative predictive value (NPV) from 10 papers related to RT-LAMP and 10 papers on the RT-LAMP-CRISPR-Cas COVID-19 diagnostic technique (21–40). We used the results to calculate the parameters needed for the comparison.

### *In silico* analysis and design of RT-LAMP primers, crRNAs, and RNA reporters.

The nucleocapsid gene (GenBank Gene ID: 43740575) of the SARS-CoV-2 virus was selected for study due to the fact that it shows a higher abundance of subgenomic mRNAs than other targets, which boosts the sensitivity of the diagnostic technique (58). Furthermore, the mutation rate found in this gene is lower than that in other targets, such as the spike gene and the ORF gene (59, 60). The target sequence was analyzed *in silico* with the aim of designing specific primers for amplification of a genetic region without any previously described mutation (N gene region, 12 to 213 bp [N2 gene]) (61). Three pairs of LAMP primers were designed using PrimerExplorer V5 software (F3-B3, FIP-BIP, and Floop-Bloop) to amplify the SARS-CoV-2 N2 gene. The FIP LAMP primer contained the T7 polymerase promoter in its sequences for the subsequent transcription step (Table 2).

**TABLE 2:** Sequences of primers, crRNAs, and reporters^*a*^

Name	Sequence	Position on gene
LAMP primers		
F3_N2	TGGACCCCAAAATCAGCG	12−29
B3_N2	GCCTTGTCCTCGAGGGAAT	195–213
FIP_N2	TGCGTTCTCCATTCTGGTTACTGCGAAATTAATACGACTCACTATAGGGAATGCACCCCGCATTACG	
BIP_N2	CGCGATCAAAACAACGTCGGCCCTTGCCATGTTGAGTGAGA	
Floop_N2	CAGTTGAATCTGAGGGTCCACCAA	50–73
Bloop_N2	CAAGGTTTACCCAATAATACTGCGT	127–151
crRNA		
crRNA_N2	gauuuagacuaccccaaaaacgaaggggacuaaaacGGUCCACCAAACGUAAUGCGGGGUGCAU	40–59
Reporters		
Reporter 1	FAM-mArArUrGrGrCmAmArArUrGrGrCmA-Biotin	
Reporter 2	FAM-UUUUUU-Biotin	

a Underlined letters indicate overhang T7 promoter sequences, and lowercase letters indicate scaffold sequences. All primers were supplied by IDT, and reporters were supplied by GenScript.

Two different RNA reporters (reporters 1 and 2) were used to reveal the results in order to select the one with the best signal. Both contained a single isomer derivative of fluorescein modification (FAM) at the 5′ extreme and a biotin molecule at the 3′ extreme (Table 2).

### Clinical samples.

Clinical samples were supplied by the Microbiology Service of the Teresa Herrera Materno Infantil Hospital (A Coruña, Spain). The samples (*n* = 133) were obtained from nasopharyngeal swabs for SARS-CoV-2 detection (Table 3).

**TABLE 3:** Positive and negative samples for SARS-CoV-2

Result	No. of samples	Origin	*C_T_* ^*a*^
Positive	27	Nasopharyngeal	<20
	48	Nasopharyngeal	20–30
	24	Nasopharyngeal	>30
Total	99		
Negative	34	Nasopharyngeal	

a *C_T_*, cycle threshold.

### Ethical approval.

Ethical approval was granted by the Galicia Drug Research Ethics Committee (CEIm-G), and internal ethical approval was received by the Institute of Research A Coruña (INIBIC) from Coruña Hospital (CHUAC) (2020/207).

### Sample processing.

For sample processing, a proteinase K-heat inactivation (PK-HID) protocol was applied to samples from swabs stored in viral transport medium (Gibco) (62) as follows. Aliquots (95 μL) of samples were treated for 15 min at 55°C with 5 μL of proteinase K (10 mg/mL; stock), prepared at 1 mg/mL in a final volume of 100 μL, and heat-inactivated at 98°C for 5 min. Finally, the extracted RNA samples were stored at −80°C.

### RT-LAMP reaction.

Amplification using the RT-LAMP (WarmStart LAMP kit [DNA and RNA]; NEB) reaction was performed following the manufacturer’s protocol. Briefly, RNA samples (5 μL) were added to a reaction mix containing 12.5 μL of WarmStart LAMP 2× master mix and 2.5 μL of 10× primer mix (FIP-BIP, 16 μM; F3-B3, 2 μM; Floop-Bloop, 4 μM; stock) adjusted to a final volume of 25 μL with dH_2_O. The reaction mixtures were incubated at 65°C for 1 h.

### Collateral-based detection.

Each Cas13a-based detection reaction mixture was incubated at 37°C for 30 min with the following reaction components: 2 μL of 10× cleavage buffer (200 mM HEPES, 90 mM magnesium chloride, 600 mM sodium chloride), 0.5 μL of deoxynucleoside triphosphates (dNTPs) (HiScribe T7 quick high-yield RNA synthesis kit), 0.5 μL of T7 polymerase (HiScribe T7 quick high-yield RNA synthesis kit), 20 U RNase murine inhibitor (NEB), 0.15 μL Cas13a endonuclease (25 nM; MCLAB), 0.5 μL crRNA (50 nM; IDT), 2 μL reporter (1,000 nM; IDT), and 5 μL of a cDNA sample, adjusted to a final volume of 20 μL with dH_2_O.

Different concentrations of Cas13a and crRNA (200, 100, and 50 nM) were tested, and two different enzyme/guide molar ratios were used (1:1 and 2:1).

### HybriDetect lateral flow assay.

Results were revealed using the HybriDetect lateral flow assay as described by the manufacturer (Milenia Biotec), with some modifications. Briefly, 20 μL of collateral-based detection product was mixed with 80 μL of assay buffer in a 96-well plate. Immediately, the gold extreme of the trip was submerged in the mix and held for 2 to 3 min.

Following the manufacturer’s instructions, the reactive strips required calibration before application for management of an optimal RNA reporter concentration, and as mentioned, reporters 1 and 2 were tested. The results obtained using two different assay buffers were also compared: the kit assay buffer and the same supplemented with 5% polyethylene glycol (PEG).

The results obtained, i.e., true positive (TP), false positive (FP), false negative (FN), and true negative (TN), were used to calculate the following parameters: sensitivity (TP/TP+FN), specificity (TN/TN+FP), PPV (TP/TP+FP), and NPV (TN/TN+FN).

### Limit of detection.

For estimating the number of initial SARS-CoV-2 viral particles that the CRISPR-Cas13a technology was able to detect, we serially diluted (1:10) the RNA extracted using hospital equipment from two clinical samples with *C_T_* values of 20 and 25. Finally, 5-μL aliquots of each dilution were used for calculation of the limit of detection (LOD). Here, we applied an estimated correlation between the *C_T_* value and the viral load.

### Statistical analysis.

Statistical analysis was conducted using the GraphPad Prism9 program to construct a receiver operating characteristic (ROC) curve with a confidence interval of 95% (Wilson/Brown method) and to construct a scatterplot of two groups (false-negative and true-positive samples) against the *C_T_* value of each sample.

## References

[1] Stasi C, Fallani S, Voller F, Silvestri C. 2020. Treatment for COVID-19: an overview. Eur J Pharmacol 889:173644. doi:10.1016/j.ejphar.2020.173644. 33053381 PMC7548059

[2] Salian VS, Wright JA, Vedell PT, Nair S, Li CX, Kandimalla M, Tang XJ, Porquera EMC, Kalari KR, Kandimalla KK. 2021. COVID-19 transmission, current treatment, and future therapeutic strategies. Mol Pharm 18:754–771. doi:10.1021/acs.molpharmaceut.0c00608. 33464914

[3] Taleghani N, Taghipour F. 2021. Diagnosis of COVID-19 for controlling the pandemic: a review of the state-of-the-art. Biosens Bioelectron 174:112830. doi:10.1016/j.bios.2020.112830. 33339696 PMC7694563

[4] Vitiello A, Ferrara F, Troiano V, La Porta R. 2021. COVID-19 vaccines and decreased transmission of SARS-CoV-2. Inflammopharmacology 29:1357–1360. doi:10.1007/s10787-021-00847-2. 34279767 PMC8287551

[5] Rai P, Kumar BK, Deekshit VK, Karunasagar I, Karunasagar I. 2021. Detection technologies and recent developments in the diagnosis of COVID-19 infection. Appl Microbiol Biotechnol 105:441–455. doi:10.1007/s00253-020-11061-5. 33394144 PMC7780074

[6] Kaminski MM, Abudayyeh OO, Gootenberg JS, Zhang F, Collins JJ. 2021. CRISPR-based diagnostics. Nat Biomed Eng 5:643–656. doi:10.1038/s41551-021-00760-7. 34272525

[7] Drame M, Tabue TM, Proye E, Hequet F, Hentzien M, Kanagaratnam L, Godaert L. 2020. Should RT-PCR be considered a gold standard in the diagnosis of COVID-19? J Med Virol 92:2312–2313. doi:10.1002/jmv.25996. 32383182 PMC7267274

[8] Cassaniti I, Novazzi F, Giardina F, Salinaro F, Sachs M, Perlini S, Bruno R, Mojoli F, Baldanti F, Members of the San Matteo Pavia COVID-19 Task Force. 2020. Performance of VivaDiag COVID-19 IgM/IgG rapid test is inadequate for diagnosis of COVID-19 in acute patients referring to emergency room department. J Med Virol 92:1724–1727. doi:10.1002/jmv.25800. 32227490 PMC7228409

[9] Goudouris ES. 2021. Laboratory diagnosis of COVID-19. J Pediatr (Rio J) 97:7–12. doi:10.1016/j.jped.2020.08.001. 32882235 PMC7456621

[10] Teymouri M, Mollazadeh S, Mortazavi H, Naderi Ghale-Noie Z, Keyvani V, Aghababaei F, Hamblin MR, Abbaszadeh-Goudarzi G, Pourghadamyari H, Hashemian SMR, Mirzaei H. 2021. Recent advances and challenges of RT-PCR tests for the diagnosis of COVID-19. Pathol Res Pract 221:153443. doi:10.1016/j.prp.2021.153443. 33930607 PMC8045416

[11] Arevalo-Rodriguez I, Buitrago-Garcia D, Simancas-Racines D, Zambrano-Achig P, Del Campo R, Ciapponi A, Sued O, Martinez-Garcia L, Rutjes AW, Low N, Bossuyt PM, Perez-Molina JA, Zamora J. 2020. False-negative results of initial RT-PCR assays for COVID-19: a systematic review. PLoS One 15:e0242958. doi:10.1371/journal.pone.0242958. 33301459 PMC7728293

[12] Zou Y, Mason MG, Botella JR. 2020. Evaluation and improvement of isothermal amplification methods for point-of-need plant disease diagnostics. PLoS One 15:e0235216. doi:10.1371/journal.pone.0235216. 32598374 PMC7323990

[13] Silva S, Pardee K, Pena L. 2019. Loop-mediated isothermal amplification (LAMP) for the diagnosis of Zika virus: a review. Viruses 12:19. doi:10.3390/v12010019. 31877989 PMC7019470

[14] Seki M, Kilgore PE, Kim EJ, Ohnishi M, Hayakawa S, Kim DW. 2018. Loop-mediated isothermal amplification methods for diagnosis of bacterial meningitis. Front Pediatr 6:57. doi:10.3389/fped.2018.00057. 29594087 PMC5857938

[15] Kashir J, Yaqinuddin A. 2020. Loop mediated isothermal amplification (LAMP) assays as a rapid diagnostic for COVID-19. Med Hypotheses 141:109786. doi:10.1016/j.mehy.2020.109786. 32361529 PMC7182526

[16] Strich JR, Chertow DS. 2019. CRISPR-Cas biology and its application to infectious diseases. J Clin Microbiol 57:e01307-18. doi:10.1128/JCM.01307-18. 30429256 PMC6440769

[17] Ding W, Zhang Y, Shi S. 2020. Development and application of CRISPR/Cas in microbial biotechnology. Front Bioeng Biotechnol 8:711. doi:10.3389/fbioe.2020.00711. 32695770 PMC7338305

[18] Chen JS, Ma E, Harrington LB, Da Costa M, Tian X, Palefsky JM, Doudna JA. 2018. CRISPR-Cas12a target binding unleashes indiscriminate single-stranded DNase activity. Science 360:436–439. doi:10.1126/science.aar6245. 29449511 PMC6628903

[19] Kellner MJ, Koob JG, Gootenberg JS, Abudayyeh OO, Zhang F. 2019. SHERLOCK: nucleic acid detection with CRISPR nucleases. Nat Protoc 14:2986–3012. doi:10.1038/s41596-019-0210-2. 31548639 PMC6956564

[20] Bhatt A, Bumbrah GS, Ruwali M, Hameed S, Fatima Z. 2022. Diagnostic efficiency of RT-LAMP integrated CRISPR-Cas technique for COVID-19: a systematic review and meta-analysis. Pathog Glob Health doi:10.1080/20477724.2022.2035625. PMC8862172 35142264

[21] Santiago-Frangos A, Hall LN, Nemudraia A, Nemudryi A, Krishna P, Wiegand T, Wilkinson RA, Snyder DT, Hedges JF, Cicha C, Lee HH, Graham A, Jutila MA, Taylor MP, Wiedenheft B. 2021. Intrinsic signal amplification by type III CRISPR-Cas systems provides a sequence-specific SARS-CoV-2 diagnostic. Cell Rep Med 2:100319. doi:10.1016/j.xcrm.2021.100319. 34075364 PMC8157118

[22] DeFina SM, Wang J, Yang L, Zhou H, Adams J, Cushing W, Tuohy B, Hui P, Liu C, Pham K. 2022. SaliVISION: a rapid saliva-based COVID-19 screening and diagnostic test with high sensitivity and specificity. Sci Rep 12:5729. doi:10.1038/s41598-022-09718-4. 35388102 PMC8986854

[23] Lai MY, Suppiah J, Thayan R, Ismail I, Mustapa NI, Soh TST, Hassan AH, Peariasamy KM, Lee YL, Lau YL. 2022. RNA purification-free detection of SARS-CoV-2 using reverse transcription loop-mediated isothermal amplification (RT-LAMP). Trop Med Health 50:2. doi:10.1186/s41182-021-00396-y. 34980275 PMC8723997

[24] Bhatt A, Fatima Z, Ruwali M, Misra CS, Rangu SS, Rath D, Rattan A, Hameed S. 2022. CLEVER assay: a visual and rapid RNA extraction-free detection of SARS-CoV-2 based on CRISPR-Cas integrated RT-LAMP technology. J Appl Microbiol 133:410–421. doi:10.1111/jam.15571. 35396760 PMC9111511

[25] Dong Y, Zhao Y, Li S, Wan Z, Lu R, Yang X, Yu G, Reboud J, Cooper JM, Tian Z, Zhang C. 2022. Multiplex, real-time, point-of-care RT-LAMP for SARS-CoV-2 detection using the HFman probe. ACS Sens 7:730–739. doi:10.1021/acssensors.1c02079. 35192340 PMC8887655

[26] Schneider FS, Molina L, Picot MC, L'Helgoualch N, Espeut J, Champigneux P, Alali M, Baptiste J, Cardeur L, Carniel C, Davy M, Dedisse D, Dubuc B, Fenech H, Foulongne V, Gaillard CF, Galtier F, Makinson A, Marin G, Santos RM, Morquin D, Ouedraogo A, Lejeune AP, Quenot M, Keiflin P, Robles FC, Rego CR, Salvetat N, Trento C, Vetter D, Molina F, Reynes J. 2022. Performances of rapid and connected salivary RT-LAMP diagnostic test for SARS-CoV-2 infection in ambulatory screening. Sci Rep 12:2843. doi:10.1038/s41598-022-04826-7. 35181680 PMC8857239

[27] Minami K, Masutani R, Suzuki Y, Kubota M, Osaka N, Nakanishi T, Nakano T, Ukimura A. 2021. Evaluation of SARS-CoV-2 RNA quantification by RT-LAMP compared to RT-qPCR. J Infect Chemother 27:1068–1071. doi:10.1016/j.jiac.2021.05.004. 34006453 PMC8112399

[28] Tang Z, Nouri R, Dong M, Yang J, Greene W, Zhu Y, Yon M, Nair MS, Kuchipudi SV, Guan W. 2022. Rapid detection of novel coronavirus SARS-CoV-2 by RT-LAMP coupled solid-state nanopores. Biosens Bioelectron 197:113759. doi:10.1016/j.bios.2021.113759. 34741956 PMC8560184

[29] Kidd SP, Burns D, Armson B, Beggs AD, Howson ELA, Williams A, Snell G, Wise EL, Goring A, Vincent-Mistiaen Z, Grippon S, Sawyer J, Cassar C, Cross D, Lewis T, Reid SM, Rivers S, James J, Skinner P, Banyard A, Davies K, Ptasinska A, Whalley C, Ferguson J, Bryer C, Poxon C, Bosworth A, Kidd M, Richter A, Burton J, Love H, Fouch S, Tillyer C, Sowood A, Patrick H, Moore N, Andreou M, Morant N, Houghton R, Parker J, Slater-Jefferies J, Brown I, Gretton C, Deans Z, Porter D, Cortes NJ, Douglas A, Hill SL, Godfrey KM, Fowler VL. 2022. Reverse-transcription loop-mediated isothermal amplification has high accuracy for detecting severe acute respiratory syndrome coronavirus 2 in saliva and nasopharyngeal/oropharyngeal swabs from asymptomatic and symptomatic individuals. J Mol Diagn 24:320–336. doi:10.1016/j.jmoldx.2021.12.007. 35121140 PMC8806713

[30] Agarwal S, Warmt C, Henkel J, Schrick L, Nitsche A, Bier FF. 2022. Lateral flow-based nucleic acid detection of SARS-CoV-2 using enzymatic incorporation of biotin-labeled dUTP for POCT use. Anal Bioanal Chem 414:3177–3186. doi:10.1007/s00216-022-03880-4. 35044487 PMC8766626

[31] Kitajima H, Tamura Y, Yoshida H, Kinoshita H, Katsuta H, Matsui C, Matsushita A, Arai T, Hashimoto S, Iuchi A, Hirashima T, Morishita H, Matsuoka H, Tanaka T, Nagai T. 2021. Clinical COVID-19 diagnostic methods: comparison of reverse transcription loop-mediated isothermal amplification (RT-LAMP) and quantitative RT-PCR (qRT-PCR). J Clin Virol 139:104813. doi:10.1016/j.jcv.2021.104813. 33848785 PMC7997201

[32] Promlek T, Thanunchai M, Phumisantiphong U, Hansirisathit T, Phuttanu C, Dongphooyao S, Thongsopa W, Nuchnoi P. 2022. Performance of colorimetric reverse transcription loop-mediated isothermal amplification as a diagnostic tool for SARS-CoV-2 infection during the fourth wave of COVID-19 in Thailand. Int J Infect Dis 116:133–137. doi:10.1016/j.ijid.2021.12.351. 34958929 PMC8709723

[33] Steens JA, Zhu Y, Taylor DW, Bravo JPK, Prinsen SHP, Schoen CD, Keijser BJF, Ossendrijver M, Hofstra LM, Brouns SJJ, Shinkai A, van der Oost J, Staals RHJ. 2021. SCOPE enables type III CRISPR-Cas diagnostics using flexible targeting and stringent CARF ribonuclease activation. Nat Commun 12:5033. doi:10.1038/s41467-021-25337-5. 34413302 PMC8376896

[34] Mahas A, Wang Q, Marsic T, Mahfouz MM. 2021. A novel miniature CRISPR-Cas13 system for SARS-CoV-2 diagnostics. ACS Synth Biol 10:2541–2551. doi:10.1021/acssynbio.1c00181. 34546709

[35] Pang B, Xu J, Liu Y, Peng H, Feng W, Cao Y, Wu J, Xiao H, Pabbaraju K, Tipples G, Joyce MA, Saffran HA, Tyrrell DL, Zhang H, Le XC. 2020. Isothermal amplification and ambient visualization in a single tube for the detection of SARS-CoV-2 using loop-mediated amplification and CRISPR technology. Anal Chem 92:16204–16212. doi:10.1021/acs.analchem.0c04047. 33238709

[36] Nguyen LT, Macaluso NC, Pizzano BLM, Cash MN, Spacek J, Karasek J, Miller MR, Lednicky JA, Dinglasan RR, Salemi M, Jain PK. 2022. A thermostable Cas12b from Brevibacillus leverages one-pot discrimination of SARS-CoV-2 variants of concern. EBioMedicine 77:103926. doi:10.1016/j.ebiom.2022.103926. 35290826 PMC8917962

[37] Ali Z, Aman R, Mahas A, Rao GS, Tehseen M, Marsic T, Salunke R, Subudhi AK, Hala SM, Hamdan SM, Pain A, Alofi FS, Alsomali A, Hashem AM, Khogeer A, Almontashiri NAM, Abedalthagafi M, Hassan N, Mahfouz MM. 2020. iSCAN: an RT-LAMP-coupled CRISPR-Cas12 module for rapid, sensitive detection of SARS-CoV-2. Virus Res 288:198129. doi:10.1016/j.virusres.2020.198129. 32822689 PMC7434412

[38] Joung J, Ladha A, Saito M, Kim NG, Woolley AE, Segel M, Barretto RPJ, Ranu A, Macrae RK, Faure G, Ioannidi EI, Krajeski RN, Bruneau R, Huang MW, Yu XG, Li JZ, Walker BD, Hung DT, Greninger AL, Jerome KR, Gootenberg JS, Abudayyeh OO, Zhang F. 2020. Detection of SARS-CoV-2 with SHERLOCK one-pot testing. N Engl J Med 383:1492–1494. doi:10.1056/NEJMc2026172. 32937062 PMC7510942

[39] Cao Y, Wu J, Pang B, Zhang H, Le XC. 2021. CRISPR/Cas12a-mediated gold nanoparticle aggregation for colorimetric detection of SARS-CoV-2. Chem Commun (Camb) 57:6871–6874. doi:10.1039/d1cc02546e. 34169944

[40] Yoshimi K, Takeshita K, Yamayoshi S, Shibumura S, Yamauchi Y, Yamamoto M, Yotsuyanagi H, Kawaoka Y, Mashimo T. 2022. CRISPR-Cas3-based diagnostics for SARS-CoV-2 and influenza virus. iScience 25:103830. doi:10.1016/j.isci.2022.103830. 35128347 PMC8801231

[41] Chu DKW, Pan Y, Cheng SMS, Hui KPY, Krishnan P, Liu Y, Ng DYM, Wan CKC, Yang P, Wang Q, Peiris M, Poon LLM. 2020. Molecular diagnosis of a novel coronavirus (2019-nCoV) causing an outbreak of pneumonia. Clin Chem 66:549–555. doi:10.1093/clinchem/hvaa029. 32031583 PMC7108203

[42] UNICEF, UNDP, World Bank, WHO Special Programme for Research and Training in Tropical Diseases, World Health Organization. 2010. Accessible quality-assured diagnostics: annual report 2009. https://apps.who.int/iris/handle/10665/84529.

[43] Hajian-Tilaki K. 2013. Receiver operating characteristic (ROC) curve analysis for medical diagnostic test evaluation. Caspian J Intern Med 4:627–635. 24009950 PMC3755824

[44] Foinquinos J, Duarte MDC, Figueiroa JN, Correia JB, Cavalcanti NV. 2021. Temporal validation of a predictive score for death in children with visceral leishmaniasis. J Trop Med 2021:6688444. doi:10.1155/2021/6688444. 34976072 PMC8716242

[45] Mendoza EJ, Manguiat K, Wood H, Drebot M. 2020. Two detailed plaque assay protocols for the quantification of infectious SARS-CoV-2. Curr Protoc Microbiol 57:ecpmc105. doi:10.1002/cpmc.105. 32475066 PMC7300432

[46] Case JB, Bailey AL, Kim AS, Chen RE, Diamond MS. 2020. Growth, detection, quantification, and inactivation of SARS-CoV-2. Virology 548:39–48. doi:10.1016/j.virol.2020.05.015. 32838945 PMC7293183

[47] Wang Q, Zhang B, Xu X, Long F, Wang J. 2018. CRISPR-typing PCR (ctPCR), a new Cas9-based DNA detection method. Sci Rep 8:14126. doi:10.1038/s41598-018-32329-x. 30237405 PMC6148268

[48] Chen JS, Ma E, Harrington LB, Da Costa M, Tian X, Palefsky JM, Doudna JA. 2021. Erratum for the report “CRISPR-Cas12a target binding unleashes indiscriminate single-stranded DNase activity.” Science 371:eabh0317. doi:10.1126/science.abh0317. 29449511 PMC6628903

[49] Pardee K, Green AA, Takahashi MK, Braff D, Lambert G, Lee JW, Ferrante T, Ma D, Donghia N, Fan M, Daringer NM, Bosch I, Dudley DM, O'Connor DH, Gehrke L, Collins JJ. 2016. Rapid, low-cost detection of Zika virus using programmable biomolecular components. Cell 165:1255–1266. doi:10.1016/j.cell.2016.04.059. 27160350

[50] Myhrvold C, Freije CA, Gootenberg JS, Abudayyeh OO, Metsky HC, Durbin AF, Kellner MJ, Tan AL, Paul LM, Parham LA, Garcia KF, Barnes KG, Chak B, Mondini A, Nogueira ML, Isern S, Michael SF, Lorenzana I, Yozwiak NL, MacInnis BL, Bosch I, Gehrke L, Zhang F, Sabeti PC. 2018. Field-deployable viral diagnostics using CRISPR-Cas13. Science 360:444–448. doi:10.1126/science.aas8836. 29700266 PMC6197056

[51] He Q, Yu D, Bao M, Korensky G, Chen J, Shin M, Kim J, Park M, Qin P, Du K. 2020. High-throughput and all-solution phase African swine fever virus (ASFV) detection using CRISPR-Cas12a and fluorescence based point-of-care system. Biosens Bioelectron 154:112068. doi:10.1016/j.bios.2020.112068. 32056963

[52] Qin P, Park M, Alfson KJ, Tamhankar M, Carrion R, Patterson JL, Griffiths A, He Q, Yildiz A, Mathies R, Du K. 2019. Rapid and fully microfluidic Ebola virus detection with CRISPR-Cas13a. ACS Sens 4:1048–1054. doi:10.1021/acssensors.9b00239. 30860365

[53] Gootenberg JS, Abudayyeh OO, Lee JW, Essletzbichler P, Dy AJ, Joung J, Verdine V, Donghia N, Daringer NM, Freije CA, Myhrvold C, Bhattacharyya RP, Livny J, Regev A, Koonin EV, Hung DT, Sabeti PC, Collins JJ, Zhang F. 2017. Nucleic acid detection with CRISPR-Cas13a/C2c2. Science 356:438–442. doi:10.1126/science.aam9321. 28408723 PMC5526198

[54] Gootenberg JS, Abudayyeh OO, Kellner MJ, Joung J, Collins JJ, Zhang F. 2018. Multiplexed and portable nucleic acid detection platform with Cas13, Cas12a, and Csm6. Science 360:439–444. doi:10.1126/science.aaq0179. 29449508 PMC5961727

[55] Zhang Y, Qian L, Wei W, Wang Y, Wang B, Lin P, Liu W, Xu L, Li X, Liu D, Cheng S, Li J, Ye Y, Li H, Zhang X, Dong Y, Zhao X, Liu C, Zhang HM, Ouyang Q, Lou C. 2017. Paired design of dCas9 as a systematic platform for the detection of featured nucleic acid sequences in pathogenic strains. ACS Synth Biol 6:211–216. doi:10.1021/acssynbio.6b00215. 27718551

[56] Quan J, Langelier C, Kuchta A, Batson J, Teyssier N, Lyden A, Caldera S, McGeever A, Dimitrov B, King R, Wilheim J, Murphy M, Ares LP, Travisano KA, Sit R, Amato R, Mumbengegwi DR, Smith JL, Bennett A, Gosling R, Mourani PM, Calfee CS, Neff NF, Chow ED, Kim PS, Greenhouse B, DeRisi JL, Crawford ED. 2019. FLASH: a next-generation CRISPR diagnostic for multiplexed detection of antimicrobial resistance sequences. Nucleic Acids Res 47:e83. doi:10.1093/nar/gkz418. 31114866 PMC6698650

[57] Ortiz-Cartagena C, Blasco L, Fernandez-Garcia L, Pacios O, Bleriot I, Lopez Diaz M, Fernandez-Cuenca F, Canton R, Tomas M. 2022. Application of RT-LAMP-CRISPR-Cas13a technology to the detection of OXA-48 producing Klebsiella pneumoniae. bioRxiv. doi:10.1101/2022.08.29.505698.

[58] Banko A, Petrovic G, Miljanovic D, Loncar A, Vukcevic M, Despot D, Cirkovic A. 2021. Comparison and sensitivity evaluation of three different commercial real-time quantitative PCR kits for SARS-CoV-2 detection. Viruses 13:1321. doi:10.3390/v13071321. 34372527 PMC8309997

[59] Majumdar P, Niyogi S. 2021. SARS-CoV-2 mutations: the biological trackway towards viral fitness. Epidemiol Infect 149:e110. doi:10.1017/S0950268821001060. 33928885 PMC8134885

[60] Thakur S, Sasi S, Pillai SG, Nag A, Shukla D, Singhal R, Phalke S, Velu GSK. 2022. SARS-CoV-2 mutations and their impact on diagnostics, therapeutics and vaccines. Front Med (Lausanne) 9:815389. doi:10.3389/fmed.2022.815389. 35273977 PMC8902153

[61] Ziegler K, Steininger P, Ziegler R, Steinmann J, Korn K, Ensser A. 2020. SARS-CoV-2 samples may escape detection because of a single point mutation in the N gene. Euro Surveill 25. doi:10.2807/1560-7917.ES.2020.25.39.2001650. PMC7531073 33006300

[62] Genoud V, Stortz M, Waisman A, Berardino BG, Verneri P, Dansey V, Salvatori M, Remes Lenicov F, Levi V. 2021. Extraction-free protocol combining proteinase K and heat inactivation for detection of SARS-CoV-2 by RT-qPCR. PLoS One 16:e0247792. doi:10.1371/journal.pone.0247792. 33635936 PMC7909620

